# Factors Influencing Triage Decisions in Patients Referred for ICU Admission

**DOI:** 10.4021/jocmr1501w

**Published:** 2013-08-05

**Authors:** Jose Orsini, Ashvin Butala, Noeen Ahmad, Alfonso Llosa, Ramesh Prajapati, Edward Fishkin

**Affiliations:** a Department of Medicine, New York University School of Medicine at Woodhull Medical and Mental Health Center, 760 Broadway, Brooklyn, NY 11206, USA

**Keywords:** Triage, Critically ill, Intensive Care Unit (ICU), Emergency Department (ED), Post-Anesthesia Care Unit (PACU), Step-down unit

## Abstract

**Background:**

Few data is available on triage of critically ill patients. Because the demand for ICU beds often exceeds their availability, frequently intensivists need to triage these patients in order to equally and efficiently distribute the available resources based on the concept of potential benefit and reasonable chance of recovery. The objective of this study is to evaluate factors influencing triage decisions among patients referred for ICU admission and to assess its impact in outcome.

**Methods:**

A single-center, prospective, observational study of 165 consecutive triage evaluations was conducted in patients referred for ICU admission that were either accepted, or refused and treated on the medical or surgical wards as well as the step-down and telemetry units.

**Results:**

Seventy-one patients (43.0%) were accepted for ICU admission. Mean Acute Physiology and Chronic Health Evaluation (APACHE)-II score was 15.3 (0 - 36) and 13.9 (0 - 30) for accepted and refused patients, respectively. Three patients (4.2%) had active advance directives on admission to ICU. Age, gender, and number of ICU beds available at the time of evaluation were not associated with triage decisions. Thirteen patients (18.3%) died in ICU, while the in-hospital mortality for refused patients was 12.8%.

**Conclusion:**

Refusal of admission to ICU is common, although patients in which ICU admission is granted have higher mortality. Presence of active advance directives seems to play an important role in the triage decision process. Further efforts are needed to define which patients are most likely to benefit from ICU admission. Triage protocols or guidelines to promote efficient critical care beds use are warranted.

## Introduction

Healthcare resource allocation refers to the distribution of healthcare resources among individuals and populations and encompasses rationing and triage. With more complex medical procedures, increasing patient age and expectations, and the increased severity of diseases, there is a greater demand for all medical services. Given all these conditions, occasionally the needs for close monitoring is required in some patients. Often, when it comes to intensive care services, demand exceeds supply leading to a rationing of ICU beds. Therefore, a method of prioritizing or triaging patients is necessary, often resulting in admission refusal [1-5]. Although recommendations for ICU triage are available, compliance with them has been shown to be suboptimal. Decisions on whether to admit a critically ill patient to ICU or not are complex, since they need to balance the potential risks and benefits for the individual patient with the limited bed availability and thus the implication for future patients [6, 7]. Ideally, patients should be admitted to intensive care if they can benefit from admission with a decreased risk of death. ICU provides sophisticated technologies and therapies by specially trained personnel, which are believed to decrease mortality. However, intensive care settings may be detrimental to patients by providing overly aggressive treatments. Extremely ill patients who will die after admission to ICU or, conversely, patients who require anticipatory monitoring who will survive even if not admitted should not be transferred to ICU [8, 9]. Unfortunately, the indications for admission to ICU remain poorly defined, and the identification of patients who can benefit from intensive care is extremely difficult.

Critical Care Medicine (CCM) consumed approximately 0.5% of the USA gross domestic product, 13% of hospital costs, and 4% of national health expenditures, generating costs of more than $55 billion in 2000. From 1985 - 2000, the number of critical care beds increased by 26% in the USA, accommodating approximately 4.4 million patients admitted to ICUs annually [10, 11]. Despite concerns over the suitability and quality of care provided in the ICU at the end of life, the number of Americans who receive ICU care at the end of life is unknown. In one study, Angus DC et al reported that approximately 20% of Americans die using ICU services [12]. The provision of intensive care is a perplexing issue for clinicians and the public. Concerns about the apparent lack of beds and the appropriateness of the patients admitted are tempered by the high cost of providing this service. When evaluating a patient with a severe acute illness, intensivists must determine the following: diagnosis, prognosis, treatment, and whether ICU admission is warranted. The answers to all these questions are related to a number of factors: number of beds available in the ICU, patient characteristics and comorbidities, and characteristics of the acute illness (severity, reversibility, and predicted quality of life after ICU discharge) [7, 13, 14]. In this study, the authors evaluated factors influencing triage decisions among patients referred for ICU admission and its impact in outcome.

## Methods

### Study design and patient population

The study has a prospective, observational design. It was conducted in a general, community inner-city hospital located in Brooklyn, New York. The hospital has a total of 346 beds distributed among general internal medicine, general surgery, psychiatry, obstetrics/gynecology, and pediatrics wards. Medical and surgical wards in our institution do not have the capabilities to treat and monitor patients in whom mechanical ventilation and/or vasopressors are required. The Medical-Surgical Intensive Care Unit (MSICU) in our hospital is a closed unit composed of 12 beds, with an annual admission average of 700 patients, and a nursing:patient ratio of 1:2 - 3. The CCM service is constituted by 5 board-certified intensivists whom provided 16-hours a day in-house coverage that includes weekends and holidays. A senior medical resident evaluates all ICU consultations, and the CCM attending is required to assess every patient for whom a consultation is requested. The CCM team received notification and approved all the admissions to the ICU. In addition to the MSICU, the hospital has a 12-beds step-down unit, and a 16-beds telemetry unit. Ethics and Palliative Care Medicine (PCM) consultation services are available at our hospital, working in close collaboration with the CCM department.

All patients, 18 years or older, in whom an evaluation for ICU admission was requested were included in the study. Triage decisions were based on the clinical determination by intensivists as well as the institutional requirements and criteria for admission to the ICU. Reasons for refusal of ICU admission at triage were recorded by using multiple-choice items, which included: patient “too well”, patient “too ill”, patient “too old”, and patient “too ill and too old”. We excluded patients who died before or during the evaluation. Data obtained from the first CCM evaluation was analyzed in patients with more than one request. Patients triaged for admission to step-down and telemetry units were considered as refused from ICU admission, since those treatment areas could not be considered as a standard ICU. Variables such as age, gender, reasons for evaluation, location from where consultation was requested, active advance directives and/or PCM evaluation at the time of triage, number of ICU beds available at the time of evaluation, APACHE-II score, and outcome at ICU and/or hospital discharge were measured. Computerized medical records were reviewed and clinical information was abstracted for each patient. Institutional Review Board approved the study.

## Results

A total of 165 patients were evaluated for ICU admission during the study period (August 1, 2012 - October 31, 2012). The locations from where consults were requested are as follows: ED (106, 64.2%), medicine wards (40, 24.2%), OR/PACU (13, 7.9%), and surgery ward (6, 3.6%). Seventy-one patients (43.0%) were accepted for admission to ICU, while 94 patients (57.0%) were triaged either to medical or surgical wards, telemetry unit, or step-down unit. Ninety-three (56.4%) of evaluated patients were males. Their median age was 58 years (19 - 97). A total of 16 patients (9.7%) had active advance directives and/or PCM evaluation or follow up at the time of the CCM evaluation. Four patients (2.4%) initially admitted to medical or surgical wards had more than one evaluation requested, and none of them were admitted to ICU after the first rejection. The total number of ICU beds available during the study period was 860 (median 6, 0 - 9). The overall mortality rate was 15.2%.

From the group of patients accepted for admission to ICU, 46 (64.8%) arrived from the ED, 19 (26.8%) from medicine wards, 4 (5.6%) from the OR/PACU, and 2 (2.8%) from the surgery ward. Most common diagnosis on admission were sepsis (20, 28.2%), respiratory failure (17, 23.9%), and gastrointestinal hemorrhage (8, 11.3%). The median age of these patients was 57 years (19 - 89). Thirty-nine patients (54.9%) were males. Three patients (4.2%) had active advance directives at the time of admission to ICU. The total number of ICU beds available at the time these patients were evaluated was 382 (median 6, 1 - 9). The total number of ICU days utilized by these patients was 187 (median 2, 1 - 21). Their mean APACHE-II score was 15.3 (0 - 36). Thirteen patients (18.3%) died in ICU. Characteristics of patients who were rejected from ICU admission are highlighted in Table 1.

**Table 1:** Characteristics of Patients Refused for ICU Admission

1) Number of patients	94 (56.9%)
2) Location:	
a) OR/PACU	9 patients (69.2%)
b) Surgery ward	4 patients (66.6%)
c) ED	60 patients (56.6%)
d) Medicine ward	21 patients (52.5%)
3) Median age: 60 years (range 20 - 97)	
a) 20 - 30 years	6 patients (6.4%)
b) 31 - 40 years	8 patients (8.5%)
c) 41 - 50 years	16 patients (17.0%)
d) 51 - 64 years	22 patients (23.4%)
e) > 65 years	42 patients (44.7%)
4) Gender	
Males	54 patients (57.4%)
Females	40 patients (42.6%)
5) Active advance directives and/or PCM evaluation or follow up at the time of evaluation	13 patients (13.8%)
6) ICU beds available during the study period when these patients were evaluated	488 (median 5, range 0 - 9)
7) Diagnosis:	
a) Respiratory failure	15 patients
b) Electrolyte imbalance	11 patients
c) Acute coronary syndrome/congestive heart failure	10 patients
d) Sepsis	8 patients
e) Drug overdose	7 patients
f) Gastrointestinal hemorrhage	6 patients
g) Hypertensive emergency	6 patients
h) Acute kidney injury	4 patients
i) Seizures	4 patients
j) Other	23 patients
8) Triage:	
a) Step-down unit	77 patients (81.9%)
b) Medicine wards	15 patients (16.0%)
c) Telemetry unit	2 patients (2.1%)
9) APACHE-II score:	mean 13.9 (range 0 - 30)
10) Reasons for refusal:	
a) Too well	81 patients (86.1%)
b) Too ill	11 patients (11.7%)
c) Too ill, too old	2 patients (2.1%)
11) Mortality	12 patients (12.8%)

## Discussion

This study examined the characteristics of patients referred for ICU admission. It provides important insights into the ICU triage process, through identification of variables influencing triage decisions and reporting physicians’ justifications for refusal of admission. To our knowledge, this is the first study related to triage of critically ill patients conducted in the New York City Health and Hospitals Corporation (HHC), the largest municipal healthcare system in the USA. It is also one of the few studies conducted in USA exploring triage of patients evaluated for ICU admission.

The percentage of patients refused ICU admission has ranged from 24-57% [1, 3, 7], but most of these studies failed to distinguish among the reasons for ICU refusal. Our rate of acceptance to ICU admission (43.0%) was above reported previously [15, 16], but lower when compared with similar studies [1, 17-19]. Not surprising, most of the accepted patients arrived from the ED. It may be explained by the large volume of patients in the ED at our institution, where an average of more than 100,000 patients are seen every year; therefore, more CCM evaluations for ICU admission would be requested. In addition, there is an increased awareness in the field of emergency medicine by triaging patients with cardio-respiratory illnesses and sepsis, since some patients admitted to general wards may require transfer to ICU within 24 - 48 hours of admission [20, 21].

Interestingly is the rejection rate in this report (57.0%), which was remarkably higher than in other studies [1, 3, 7]. This high refusal rate may be related to the inclusion of all patients for whom ICU admission was requested, and probably a consequence of our institutional policies of not allowing ventilators and/or vasoactive drugs in general wards thus intensivists have to reserve ICU beds for the most critically ill patients. It also highlights the importance of organizational strategies designed to improve ICU receptivity through more appropriate use of resources. Alternatives to ICU admission are needed for patients who need stabilization or whose illnesses are too complex for general wards. Although their cost-effectiveness has not been demonstrated, intermediate care units offer theoretical advantages for lower risk patients needing monitoring and less intense nursing care, and that would give intensivists flexibility during the triage process resulting in more appropriate use of ICU beds [22]. It may be the explanation for the large number of patients (77, 81.9%) not accepted for ICU admission that was triaged to step-down unit, where our nursing:patient ratio is 1:4 - 5. Similar to data published by Cohen RI et al [17], in this study patients were rejected either for being not critical enough to justify ICU admission (“too well”; 81, 86.2%), or for having low life expectancy due to acute or chronic illness even with intensive therapy (“too ill”; 13, 13.8%). Intensive care is usually regarded as expensive, and as a result beds are limited. This has raised serious questions about rationing when there are insufficient beds for all those patients referred. In previous studies, low ICU bed availability was associated with high ICU admission refusal rates [1, 23, 24]. In our study, rejections from ICU admission were not independently associated to the availability of beds at the time of triage, since our ICU was completely occupied only in 4 opportunities during the study period.

Decisions regarding admissions/refusals to ICU admission of surgical patients can potentially strain the relationship between the critical care team and the primary surgical service. In contrast with results from other studies [1, 7, 25], surgical patients were more likely to be refused from ICU admission. From the total of 19 surgical patients evaluated, 2 died (11.5%). This result may highlight the value of step-down units managed by trained intensivists, supported by a multidisciplinary team that makes arbitration decisions safe for surgical patients. It also illustrates the possibility of monitoring and treating high-risk surgical patients in locations other than ICUs, such as PACUs. The PACU is primarily established to minimize the incidence of complications immediately after anesthesia and surgery, while providing the best quality of safe care. Our study suggests that PACU may function as a temporary ICU or step-down unit, giving its monitoring capabilities. However, PACUs were neither designed, staffed nor equipped to serve as an ICU.

One factor independently associated with rejecting admission to ICU was the presence of active advance directives. Contrasting to previous reports [2], the presence of advance directives and/or PCM involvement in patient care may have played a role in triage decisions. According to our results, and similar to previously published data [17, 26], patients with active advance directives were less likely to be admitted to the ICU. In this study, triage decisions were not independently associated with age or gender. Twenty-three patients (32.4%), above 65 years of age, were admitted and treated in ICU. This could be interpreted as that our triaging intensivists did not perceive advance age per se as a reason for refusal of ICU admission. One of the main findings in this study is that severity of illness, expressed by the APACHE-II score, was not independently associated with ICU admission or refusal decisions. Contrary to previously published data where patients with elevated APACHE-II score were likely to be refused from ICU admission [1], our study showed no significant difference in the APACHE-II score of accepted and refused patients.

As majority of ICUs in USA, the CCM division at our institution has written criteria for admission. However, as has been published, majority of intensivists do not follow strictly these criteria to make daily ICU admission decisions. Rather, decisions whether to admit or not patients to ICU often rest on the judgment of the consulting CCM physician, and there are not much data available about how such decisions are made [2]. Intensivists recognize that more care does not necessarily represent better care. Balancing patients’ needs for critical care with the available critical care resources is a great challenge. The decision to refuse ICU admission does not necessarily mean that death is considered inevitable. The refusal is justified either by the lack of benefit for the individual patient, or on the basis of the distributive justice concept. The unexpected high rate of survival (87.2%) at hospital discharge among those patients for whom admission to ICU was refused was alarming. This may be a consequence of some patients refused on the basis of a clinical perception of the triaging physician (group of “too well” patients), and other patients refused based on qualitative futility who might had some chance of surviving despite ICU care being considered unnecessary and/or futile (group of “too ill” patients).

The evidence for the cost-effectiveness of intensive care is weak, and the available data implied that those patients who are not admitted would not survive. Those randomized studies of effectiveness are difficult to justify on ethical grounds. The daily cost of intensive care has been described, but randomized controlled studies to assess cost-effectiveness of admission to ICU are unlikely to be feasible. Our results showed a lower overall mortality rate than previous studies [1]. When compared to data from other reports [27-30], mortality rate in patients admitted to ICU was lower. Furthermore, the mortality rate of patients that were refused for ICU admission was lower when compared to the patients admitted. The median age for those refused patients who did not survive was 66 years (37 - 95), majority were females (7, 58.3%), and their median APACHE-II score was 21.5 (5 - 30). Six patients (7.4%) deemed “too well” to require ICU admission died, which is comparable to data previously reported [15, 22]. It may be interpreted as that all patients referred for consideration of admission to ICU should be considered as high risk. On the other hand, the mortality rate in patients considered “too ill” to benefit from ICU admission (6, 46.2%) was lower than previously reported [28]. From the 13 patients who died in ICU, 5 (38.5%) were admitted from the hospital wards. At the time of ICU admission their mean length of stay on the wards was 9.8 days (1 - 37), which is comparable to data reported in one study [17]. Many serious events in hospitals are proceeding by hours or days of slow deterioration, resulting in multiorgan dysfunction and potentially preventable admission to the ICU. The care of the deteriorating patient in hospital wards is a global problem in most of healthcare systems, and their hospital mortality is high. Institutional, educational policies reinforcing the appropriate and effective use of the concept “ICU without walls”, practice in the form of rapid response systems, would be beneficial in order to identify and respond to serious illnesses at early stages. However, in a large prospective study, ICU admissions from hospital wards that came through a rapid response team (RRT) call resulted in higher ICU mortality [31]. Probably, this may be traduce as that ICU triage should be targeted at maximizing ICU benefit by early admitting patients deteriorating on hospital wards. Contrasting to data from other reports [30, 32, 33], this study failed to demonstrate the beneficial effect of decreasing mortality by using ICU resources, since patients treated in ICU had higher mortality rate. Unfortunately, this study does not record follow up data post-hospital discharge. However, the purpose of this study was not to determine whether triage decisions in our ICU were appropriate or not. Our purpose was to identify or associate explicit and implicit factors that might influence triage decisions in patients referred for ICU admission. Strengths of our study include its prospective design, with evaluation of all consecutive ICU triages over the study period by a dedicated CCM consultation team. However, triage decisions were made in some patients without the direct screening of a CCM team member. Therefore, some patients may have been admitted to ICU if the CCM evaluation was requested overnight. The study has some limitations. It was conducted in a single institution, and our findings may not be applicable to other ICUs with varying staffing models. Some patients may have been admitted to ICU for reasons other than clinical benefit. Those factors are difficult to measure and include the inability of healthcare workers to deliver appropriate care on hospital wards, pressure from families or other physicians, and concerns about legal actions. Despite the large number of consecutive patients, the study may have been underpowered, and a larger sample size obtained by extending the study period would perhaps have revealed additional significant differences.

### Conclusion

As critical care becomes more expensive, the need to optimize triage and rationing decisions will intensify. Critical care providers have little evidence available when seeking guidance about which patients are most likely to benefit from critical care. ICU refusal rates differ greatly across ICUs and are usually dependent on both patient characteristics and organizational factors. In ICUs staffed continuously by intensivists who evaluated every potential admission, factors other than severity of illness may be important in the triage decisions. Refusal of ICU admission is common and challenging, but alternatives locations outside the ICUs in which care for stable critically ill patients could be delivered, such as step-down units, progressive care units (PCUs), and PACUs, would allow CCM physicians a more rational distribution of ICU beds.
